# Coincident Detection of Human Papillomavirus (HPV) in the Oral and Genital Regions in a Young Adult Male with Oral Lichen Planus: A Case Report

**DOI:** 10.3390/ijms27209240

**Published:** 2026-10-18

**Authors:** Andjelija Petrović, Željko Popović, Biljana Pokimica, Vesna Vučić, Miloš Čanković

**Affiliations:** 1 Group for Nutritional Biochemistry and Dietology, Institute for Medical Research, National Institute of the Republic of Serbia, University of Belgrade, 11000 Belgrade, Serbiavesna.vucic@imi.bg.ac.rs (V.V.); 2 Department of Biology and Ecology, Faculty of Sciences, University of Novi Sad, 21000 Novi Sad, Serbia; zeljko.popovic@dbe.uns.ac.rs; 3 Centre of Research Excellence in Nutrition and Metabolism, Institute for Medical Research, National Institute of the Republic of Serbia, University of Belgrade, 11000 Belgrade, Serbia; 4 Faculty of Medicine, University of Novi Sad, Hajduk Veljkova 3, 21000 Novi Sad, Serbia; 5 Oral Medicine Section, Dentistry Department, Clinic for Dentistry of Vojvodina, Hajduk Veljkova 12, 21000 Novi Sad, Serbia

**Keywords:** oral lichen planus, human papillomavirus, co-infection, malignant transformation, young male

## Abstract

Oral lichen planus (OLP) is an atypical presentation in young males, and its co-occurrence with human papillomavirus (HPV) raises clinical concerns regarding malignant transformation. This case report describes a 27-year-old sexually active male with OLP and potentially persistent, synchronous oral and multigenotypic genital HPV co-infections. A 27-year-old male presented for follow-up 19 months after surgical excision of an oral papilloma with lichenoid features and histopathologically confirmed OLP. Molecular detection and genotyping were performed using oral and genital swabs via CFX96 real-time PCR. Results revealed potentially persistent oral co-infection with high-risk HPV 31 and low-risk HPV 44. Genital screening detected these same oral genotypes alongside four additional strains, consisting of high-risk HPV 18, 35, and 73 (classified per IARC standards), and low-risk HPV 81, with clinically evident condylomata acuminata. The long-term detection of high-risk HPV 31 in the oral swab in the presence of OLP highlights a hypothetical “dual-hit” oncogenic framework, where inflammation-induced oxidative stress may synergize with viral E6 and E7 oncoproteins to disrupt cell cycle regulation and elevate malignant transformation risks, though this remains entirely speculative in the absence of mechanistic biomarkers. Synchronous oral and anogenital HPV co-infections in young patients with OLP warrant a comprehensive multidisciplinary diagnostic protocol and cautious oncologic surveillance recommendations to prevent malignant progression and further transmission.

## 1. Introduction

Oral lichen planus (OLP) is a chronic, immune-mediated disease of the oral mucosa whose etiology remains a major research focus at the intersection of autoimmune responses and exogenous viral triggers [1,2]. Epidemiologically, OLP occurs in the general population with a prevalence ranging from 0.5% to 2%, exhibiting a marked female predilection (2:1 ratio) and most frequently manifesting during middle age [3,4]. Consequently, the presentation of confirmed OLP in a younger, 27-year-old sexually active male constitutes an uncommon clinical entity, underscoring the necessity for a more comprehensive investigation of external etiologic factors. Contemporary literature increasingly highlights the role of human papillomavirus (HPV) infection in the pathogenesis and progression of OLP. Although the primary pathomechanism of OLP is driven by an autotoxic T-lymphocyte response (CD8+ T cells) that induces basal keratinocyte apoptosis, chronic inflammation and a compromised epithelial barrier create a favorable microenvironment for HPV entry, local retention, and mucosal tissue interaction [5]. A recent meta-analysis confirmed that HPV infection is associated with an elevated risk of developing OLP, with the prevalence of high-risk genotypes (hr-HPV) being significantly higher in OLP lesions compared to healthy oral mucosa [3]. A distinct clinical and diagnostic challenge in sexually active young patients is the synchronous detection of hr-HPV genotypes in both the oral cavity and the anogenital region. The transmission of hr-HPV via orogenital practices facilitates co-infection across both anatomical sites, necessitating a coordinated multidisciplinary approach and prompt referral for anogenital evaluation by appropriate specialists to ensure the timely identification of subclinical or mucosal changes. From the perspective of oncologic surveillance, the coexistence of OLP and hr-HPV carries a potential risk of malignant transformation [6]. The World Health Organization (WHO) classifies OLP as an oral potentially malignant disorder (OPMD), with an average rate of progression to oral squamous cell carcinoma (OSCC) ranging between 1.1% and 1.4% [7]. The presence of oncogenic hr-HPV genotypes within an inflammatorily altered OLP epithelium may exert a synergistic effect that accelerates dysplastic changes. The objective of this paper is to present an atypical, rare clinical case of oral lichen planus in a 27-year-old sexually active male patient with real-time PCR-confirmed hr-HPV co-infection in both the oral cavity and the anogenital region, emphasizing the importance of a comprehensive diagnostic protocol, risk-stratified screening, and structured longitudinal surveillance [8].

## 2. Case Presentation

This case report has been drafted in strict accordance with the CARE (CAse REport) guidelines. This study was approved by the Ethics Committee of the Clinical Center of Vojvodina (approval No. 00-20/654) and was conducted in full accordance with ethical principles and the Declaration of Helsinki. A chronological overview of the patient’s clinical evaluation, diagnostic procedures, and findings is structured in the timeline below (Table 1). A 27-year-old man presented to the Clinic of Dentistry of Vojvodina, Novi Sad, for a follow-up 19 months after the surgical excision of an oral papilloma located on the right lateral border of the tongue and histopathological confirmation of two diagnoses at the site: *Papilloma mucosae cum mucositis chronica lichenoides* and *Lichen planus mucosae*.

**Figure 1:**
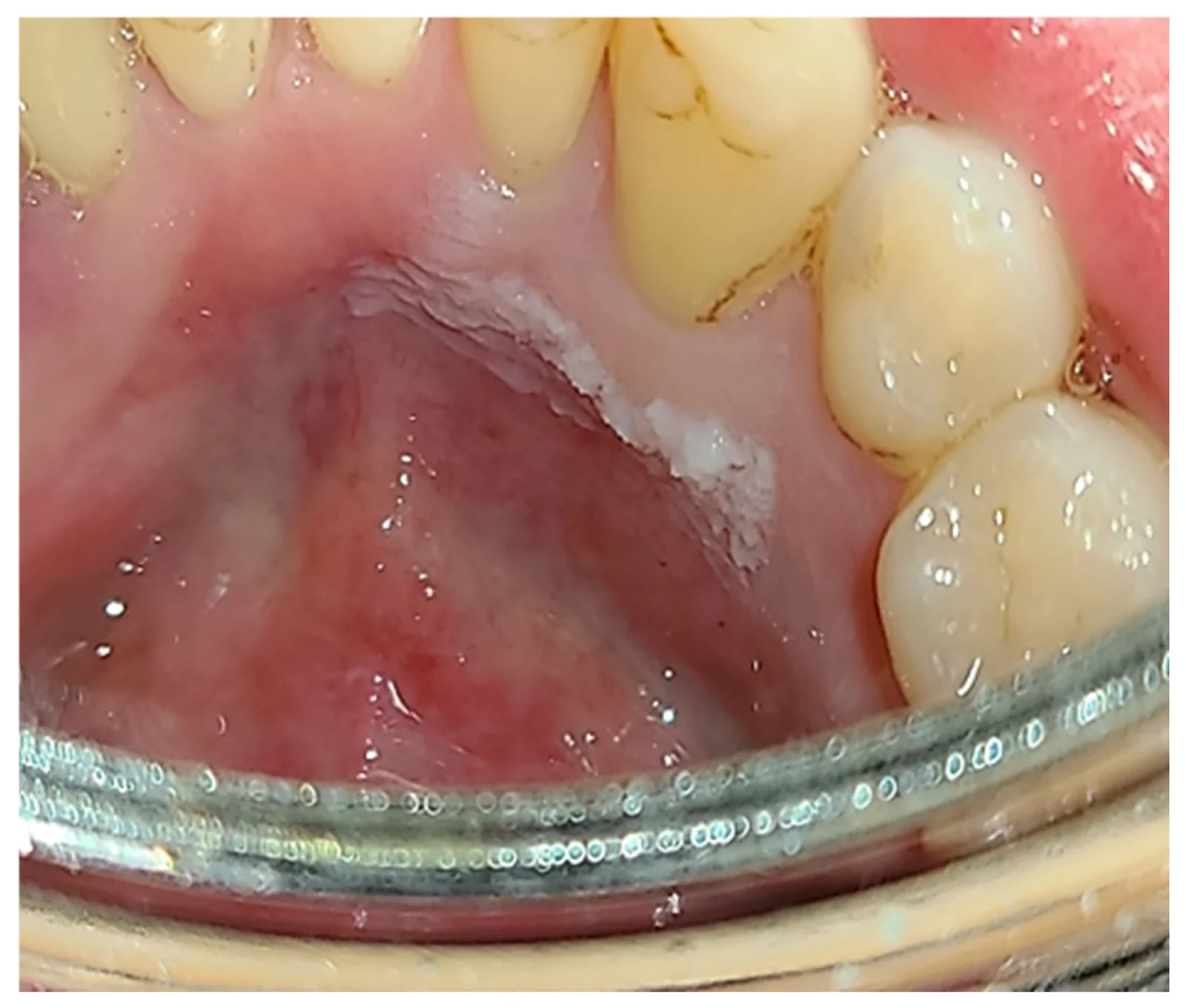
The location of the oral papilloma.

**Figure 2:**
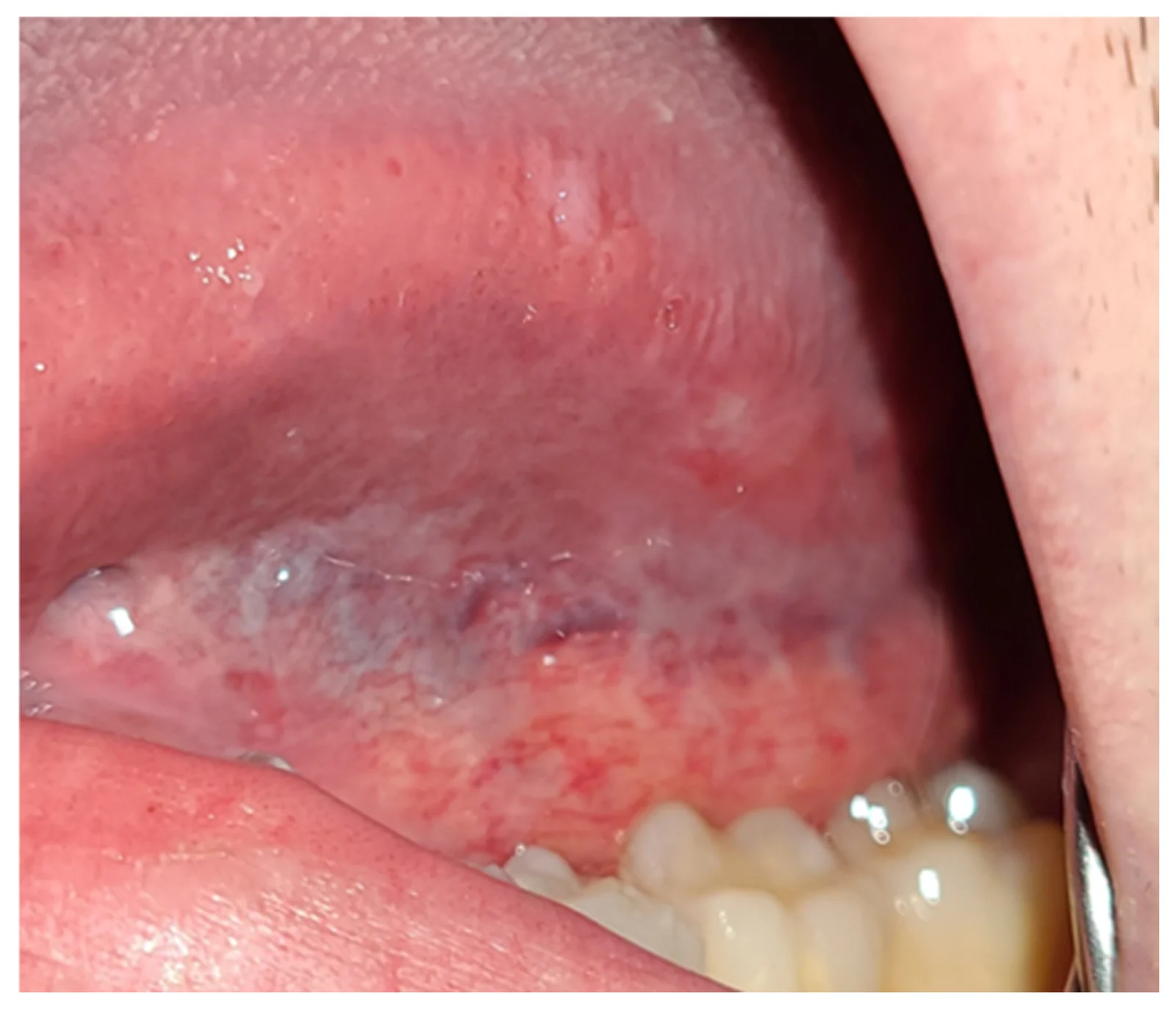
The location of oral lichen planus.

Although the histopathological diagnosis was confirmed by the pathology report, the corresponding histopathological images were not available for inclusion, which may be considered a limitation of this report. Specifically, the excised papilloma was located on the lingual side of the attached gingiva in the region of teeth 33 to 31 (Figure 1). Concurrently, the lesions corresponding to oral lichen planus (OLP) were localized on the left ventral surface of the tongue and the right buccal mucosa (Figure 2). Histopathological evaluation of the excised specimen confirmed characteristic features of OLP with the complete absence of epithelial dysplasia. At that initial evaluation, HPV genotype 31 was detected in both the oral swab and the excised papilloma tissue. The patient’s medical history revealed that he was a former smoker and sexually highly active, with his first sexual intercourse occurring at the age of 14 years. He reported engaging in reciprocal oral sex and identified as heterosexual. Regarding sexually transmitted infections, he reported a previous episode of gonococcal infection at the age of 16 years. The patient’s clinical history confirmed that he was unvaccinated against HPV. Clinical examination at the 19-month follow-up did not reveal clinically remarkable changes in oral cavity and the patient reported no subjective symptoms or complaints. A buccal mucosal swab was collected for molecular detection of 28 HPV genotypes. In accordance with multidisciplinary management recommendations for such cases, the patient was referred for screening and evaluation of the genital region. Genital sampling was performed specifically from the glans penis, with clinical condylomata observed incidentally during the sampling procedure. The oral swab tested positive for HPV genotypes 31 and 44. The genital swab revealed the same HPV genotypes detected in the oral cavity (HPV 31 and HPV 44), along with four additional HPV genotypes: HPV 18, HPV 35, HPV 73, and HPV 81.

## 3. Discussion

The presented case of a 27-year-old male patient diagnosed with OLP and a concurrent potentially persistent oral and multigenotypic genital HPV infection represents an atypical and clinically significant entity. Considering that OLP primarily affects middle-aged and older populations with a female predilection, the onset of this disease in a male in his third decade of life necessitates a thorough evaluation of potential triggers and the complex interaction between viral pathogens, mucosal immunity, and the pathogenesis of oral potentially malignant disorders [9,10,11,12].

### 3.1. Concordance Between Oral and Anogenital HPV Swabs and Transmission Dynamics

The demonstrated concordance of HPV positivity across both anatomical sites correlates with sexual activity patterns and orogenital contact. Rather than representing an autoimmune process or a direct mechanical vector transmission, the presence of identical genotypes in both sites indicates synchronous exposure or autoinoculation. In young, immunocompetent patients, oral HPV infections usually resolve spontaneously within 12 to 24 months, but persistent retention on pathologically altered mucosa serves as a notable factor in downstream mucosal changes. The demonstrated concordance of HPV positivity across both the oral cavity and the genital region in this patient strongly correlates with sexual activity patterns and orogenital contact. Epidemiological data indicate that potentially persistent oral HPV infection is primarily sexually acquired [13,14,15]. The detection of identical genotypes in both anatomical sites suggests either autoinoculation or synchronous exposure during sexual encounters, demonstrating synchronous concordance rather than proving direct vector transmission or autoinoculation definitively. In young, immunocompetent patients, the majority of oral HPV infections are transient, resolving spontaneously within 12 to 24 months. While the immune system successfully clears most HPV infections, potentially persistent viral retention can lead to sustained mucosal changes [16,17]. Persistent viral presence on traumatized or pathologically altered mucosa serves as a notable factor in downstream mucosal alterations [18,19,20]. Fantozzi et al. showed in their study that patients with high-risk HPV infections presented with oral lichen planus (40.0%) among other oral lesions [21]. In contrast to the classical association of OLP exclusively with hr-HPV genotypes 16 and 18, molecular analysis in our patient identified a dual presence of high-risk HPV-31 and low-risk HPV-44 [22]. The identification of genotypes 31 and 44 in both anatomical regions indicates a potential epidemiological link [23]. Furthermore, the broad spectrum of HPV genotypes in the genital region (encompassing high-risk strains 18, 31, 35, 73, and low-risk strains 44, 81) points to extensive pathogen exposure. Genotype 18, detected on the genital mucosa, is recognized as a high-risk oncogenic strain with strong mucosal affinity, requiring attentive clinical surveillance [24,25].

### 3.2. Malignant Potential and the Hypothetical “Dual-Hit” Mechanism

The evaluation of oncogenic potential remains a primary clinical consideration. The WHO classifies OLP as an OPMD [26]. Malignant progression of OLP to OSCC is a recognized clinical concern, with reported transformation rates varying across studies [27]. Currently, no absolute clinical or histopathological criteria exist to predict with certainty which individual lesions will undergo malignant shift. A key finding in this report is the 19-month detection of high-risk genotype HPV 31. The previous detection of this genotype in an oral swab and lesional tissue, alongside its presence in current swabs, suggests potential persistence or re-infection, as intermediate longitudinal sampling was not performed. Long-term viral persistence is recognized as an important risk factor in epithelial transformation pathways. The underlying pathological continuum unfolds slowly across extended timeframes, driven by a functional transition from productive infection to transforming viral activity. Genomic integration of viral DNA during this transition can drive the overexpression of viral oncoproteins, disrupting cell cycle regulation and promoting genomic instability. However, disease clearance remains achievable, as not all persistent cases advance to high-grade lesions [28].

Chronic inflammation in OLP induces oxidative stress and the release of reactive oxygen and nitrogen species, leading to DNA damage in keratinocytes [29]. Concurrently, E6 and E7 oncoproteins produced by hr-HPV genotypes can interact with tumor-suppressor pathways (such as p53 and pRb), impairing normal cell cycle checkpoints and DNA repair [30]. The concomitant presence of persistent hr-HPV within an active OLP lesion has been proposed in the literature as a purely hypothetical “dual-hit” mechanism for malignant transformation into OSCC, where inflammation-driven genomic instability and viral oncoprotein-mediated cell-cycle dysregulation may act synergistically; however, this model remains entirely speculative in our case due to the absence of direct mechanistic markers (such as E6/E7 mRNA expression, viral integration analysis, p16INK4a, or p53/pRb immunohistochemistry).

### 3.3. Clinical Implications and a Multidisciplinary Approach

Our case highlights several relevant clinical insights. Directing the patient to genital swab testing based on a positive oral finding proved valuable in identifying an undetected regional reservoir of high-risk HPV strains (including HPV 18 and 35). From a clinical standpoint, this demonstrates that oral mucosal findings can occasionally serve as indicators prompting broader regional screening. Beyond individual prognosis, interdisciplinary collaboration plays a useful role in managing complex viral co-infections. Consequently, our findings support the implementation of an integrated interdisciplinary referral protocol for young patients presenting with atypical oral lesions, for whom structured, risk-stratified clinical follow-up is recommended.

## 4. Materials and Methods

### 4.1. Patient Preparation and Sample Collection

Before sample collection, the patient was instructed to refrain from eating and drinking, except for water, and from smoking for at least 30 min. An oral swab was collected from the lesion using a sterile dry swab and immediately placed in transport medium. The sample was stored at −20 °C until DNA extraction.

### 4.2. DNA Extraction

DNA was extracted from the clinical specimen using the RealBest DNA-Express CE IVD extraction kit (C-8899; AO Vector-Best, Novosibirsk, Russia; BIORON GmbH, Römer-berg, Germany), according to the manufacturer’s instructions. Briefly, 100 μL of transport medium containing exfoliated cells was transferred to the lysis buffer and incubated at 98 °C for 10 min. After cooling, the sample was centrifuged at 8000 rpm for 15 min, and the resulting supernatant containing extracted DNA was used for PCR analysis.

### 4.3. HPV Detection and Genotyping

HPV DNA detection and genotyping were performed using the Freeze-dried Human Papillomavirus 28 Types Genotyping Detection Kit (Jiangsu Macro & Micro-Test Med-Tech Co., Ltd., Nantong, China), according to the manufacturer’s instructions. The assay ena-bles qualitative detection and genotyping of 28 HPV types: 6, 11, 16, 18, 26, 31, 33, 35, 39, 40, 42, 43, 44, 45, 51, 52, 53, 54, 56, 58, 59, 61, 66, 68, 73, 81, 82, and 83.

### 4.4. Real-Time PCR Procedure

The assay was performed in eight multiplex real-time PCR reactions using FAM, HEX, ROX, and Cy5 fluorescence channels. Each reaction included an internal control to monitor amplification validity. For each reaction, 20 μL of reconstituted reaction mixture was combined with 10 μL of extracted DNA, blank control, or the corresponding positive control, resulting in a final volume of 30 μL. The mixtures were vortexed for 30 s and briefly centrifuged to remove air bubbles.

Real-time PCR was performed using a CFX96 real-time PCR detection system (Bio-Rad, Hercules, CA, USA). The thermal cycling conditions were as follows: 50 °C for 2 min, 95 °C for 10 min, 10 cycles of 95 °C for 15 s and 62 °C for 20 s, followed by 30 cycles of 95 °C for 15 s and 58 °C for 31 s, with fluorescence acquisition during the final step. Positivity criteria and quantification cycle (Cq) cut-off thresholds were applied in strict accordance with the manufacturer’s analytical guidelines, verifying valid exponential amplification curves and appropriate internal control performance.

### 4.5. Quality Control and Interpretation of Results

Each PCR run included a blank control, positive controls, and an internal control. The PCR run was considered valid when all control reactions met the manufacturer’s acceptance criteria.

## 5. Conclusions

This case highlights an atypical presentation of oral lichen planus associated with synchronous oral and anogenital hr-HPV co-infection in a young adult male. Given the short 19-month follow-up period, no definitive conclusions regarding long-term oncogenic risk can be drawn. Our findings underscore the relevance of considering hr-HPV infection in atypical presentations of oral lichen planus and emphasize the utility of cross-specialty evaluation and close collaboration between dental and medical specialists. Specifically, recommending a coordinated multidisciplinary approach is valuable for early identification, appropriate clinical monitoring, and patient counseling regarding HPV transmission risks.

## Figures and Tables

**Table 1:** Case Timeline and Chronological Summary.

Timepoint	Clinical Findings and Interventions	HPV Genotypes Detected	Histopathological Status
Initial presentation	Surgical excision of oral papilloma on the right lateral border of the tongue; initial clinical evaluation.	Oral Swab: HPV 31 Papilloma Tissue: HPV 31	Papilloma mucosae cum mucositis chronica lichenoides and oral lichen planus mucosae (no dysplasia)
Interim period	Follow-up interval (19 months): conservative monitoring.	Not tested	N/A
19-Month Follow-up	Clinical re-evaluation of the lesions Referral for anogenital screening: Genital condylomata acuminata	Oral Swab: HPV 31, HPV 44 Genital Swab: HPV 18, 31, 35, 44, 73, 81	N/A

## Data Availability

The original contributions presented in this study are included in the article. Further inquiries can be directed to the corresponding author.

## Abbreviations

The following abbreviations are used in this manuscript:

HPV	Human papillomavirus
hr-HPV	High-risk human papillomavirus genotypes
OLP	Oral lichen planus
OPMD	Oral potentially malignant disorder
OSCC	Oral squamous cell carcinoma
WHO	World Health Organization
